# Association of Killer Cell Immunoglobulin-Like Receptor Genes with Hodgkin's Lymphoma in a Familial Study

**DOI:** 10.1371/journal.pone.0000406

**Published:** 2007-05-02

**Authors:** Caroline Besson, Sophie Roetynck, Fionnuala Williams, Laurent Orsi, Corinne Amiel, Catherine Lependeven, Guillemette Antoni, Olivier Hermine, Pauline Brice, Christophe Ferme, Patrice Carde, Danielle Canioni, Josette Brière, Martine Raphael, Jean-Claude Nicolas, Jacqueline Clavel, Derek Middleton, Eric Vivier, Laurent Abel

**Affiliations:** 1 Laboratoire de Génétique Humaine des Maladies Infectieuses, INSERM, U550, Paris, France; 2 Université Paris Descartes, Faculté de Médecine René Descartes, Paris, France; 3 Assistance Publique-Hôpitaux de Paris (AP-HP), Service d'Hématologie et Immunologie Biologiques, CHU Bicêtre, Le Kremlin-Bicêtre, France; 4 Université Paris Sud, Faculté de Médecine, Le Kremlin-Bicêtre, France; 5 Centre d'Immunologie de Marseille-Luminy, Université de la Méditerranée, Marseille, France; 6 INSERM, U631, Marseille, France; 7 CNRS, UMR6102, Marseille, France; 8 Northern Ireland Regional Histocompatibility and Immunogenetics Laboratory, City Hospital, Belfast, Northern Ireland; 9 INSERM, U754, Université Paris XI, Villejuif, France; 10 Laboratoire de Virologie, Hôpital Tenon, Paris, France; 11 Service d'Hématologie adultes, Hôpital Necker, Paris, France; 12 Service d'Onco-hématologie, Hôpital Saint-Louis, Paris, France; 13 Département d'Hématologie, Institut Gustave Roussy, Villejuif, France; 14 Service d'anatomo-pathologie, Hôpital Necker, Paris, France; 15 Service d'anatomo-pathologie, Hôpital Saint-Louis, Paris, France; 16 School of Biomedical Sciences, University of Ulster, Coleraine, Northern Ireland; 17 Assistance Publique–Hôpitaux de Marseille, Hôpital de la Conception, France; Rockefeller University, United States of America

## Abstract

**Background:**

Epstein-Barr virus (EBV) is the major environmental factor associated with Hodgkin's lymphoma (HL), a common lymphoma in young adults. Natural killer (NK) cells are key actors of the innate immune response against viruses. The regulation of NK cell function involves activating and inhibitory Killer cell Immunoglobulin-like receptors (KIRs), which are expressed in variable numbers on NK cells. Various viral and virus-related malignant disorders have been associated with the presence/absence of certain *KIR* genes in case/control studies. We investigated the role of the *KIR* cluster in HL in a family-based association study.

**Methodology:**

We included 90 families with 90 HL index cases (age 16–35 years) and 255 first-degree relatives (parents and siblings). We developed a procedure for reconstructing full genotypic information (number of gene copies) at each *KIR* locus from the standard *KIR* gene content. Out of the 90 collected families, 84 were informative and suitable for further analysis. An association study was then carried out with specific family-based analysis methods on these 84 families.

**Principal Findings:**

Five *KIR* genes in strong linkage disequilibrium were found significantly associated with HL. Refined haplotype analysis showed that the association was supported by a dominant protective effect of *KIR3DS1* and/or *KIR2DS1*, both of which are activating receptors. The odds ratios for developing HL in subjects with at least one copy of *KIR3DS1* or *KIR2DS1* with respect to subjects with neither of these genes were 0.44[95% confidence interval 0.23–0.85] and 0.42[0.21–0.85], respectively. No significant association was found in a tentative replication case/control study of 68 HL cases (age 18–71 years). In the familial study, the protective effect of *KIR3DS1*/*KIR2DS1* tended to be stronger in HL patients with detectable EBV in blood or tumour cells.

**Conclusions:**

This work defines a template for family-based association studies based on full genotypic information for the *KIR* cluster, and provides the first evidence that activating KIRs can have a protective role in HL.

## Introduction

Hodgkin's lymphoma (HL) differs from other lymphomas in terms of both specific pathological and epidemiological features. HL is characterised by the presence of large tumour cells known as Hodgkin and Reed-Sternberg cells, derived from a germinal centre B cell [1]. The incidence of HL displays an unusual age distribution, with two peaks—between the ages of 15 to 34 years and over the age of 60 years in most Western countries, but in children and in the oldest age groups in developing countries [2]. HL is one of the most common forms of lymphoma occurring in young adults in developed countries, with an annual incidence of around 3 per 100,000 [2], [3]. Both genetic and environmental factors are thought to be involved in the pathogenesis of HL [2]. There is growing evidence to suggest a genetic predisposition to HL, based on many reports of familial aggregation of the disease [4]–[6], including a twin study [7]. Interestingly, a review of these studies found that familial HL lacked the classic bimodal age distribution, with only one peak observed, between the ages of 15 and 34 years [6]. Several variants of the major histocompatibility complex (MHC) region have been reported to be associated with HL [8]–[10], but there is no consensus on the role of specific human leukocyte antigen (HLA) alleles or haplotypes in HL. In the only genome-wide scan by linkage analysis conducted to date, suggestive evidence was obtained for a HL susceptibility locus on chromosome 4p16 [11]. The genetic basis of HL thus remains elusive.

Several findings strongly suggest that Epstein-Barr virus (EBV) is a major environmental factor contributing to oncogenesis in HL [12], [13]. EBV clonal DNA is identified in the Reed-Sternberg cells in around 30% of cases of HL, and EBV infection is thought to provide survival signals for these abnormal B cells, leading to their proliferation [14]. Moreover, epidemiological studies have clearly shown that the risk of developing HL be up to three times higher in subjects with a previous history of infectious mononucleosis—the symptomatic form of primary EBV infection, particularly frequent in adolescence—than in other subjects [15]. HL patients have also been found to have high EBV antibody titres at the time of HL diagnosis, and years before and after diagnosis [16]. All these findings suggest that impairment of the immune response to EBV infection may contribute to the pathogenesis of HL. Natural killer (NK) cells are key actors of the innate immune response to viruses [17] [18], including EBV [19], [20]. Their role is illustrated by the recent report of a child who developed an EBV-driven lymphoproliferative disorder associated with a novel specific NK cell deficiency [21]. Further support for an involvement of these cells in innate immunity to viruses has been provided by experimental models as susceptibility to murine cytomegalovirus (MCMV), another herpes-virus, is controlled by a single gene, *Ly49H* (also called *Klra8*) encoding an NK activating receptor in mice [22], [23].

**Figure 1:**
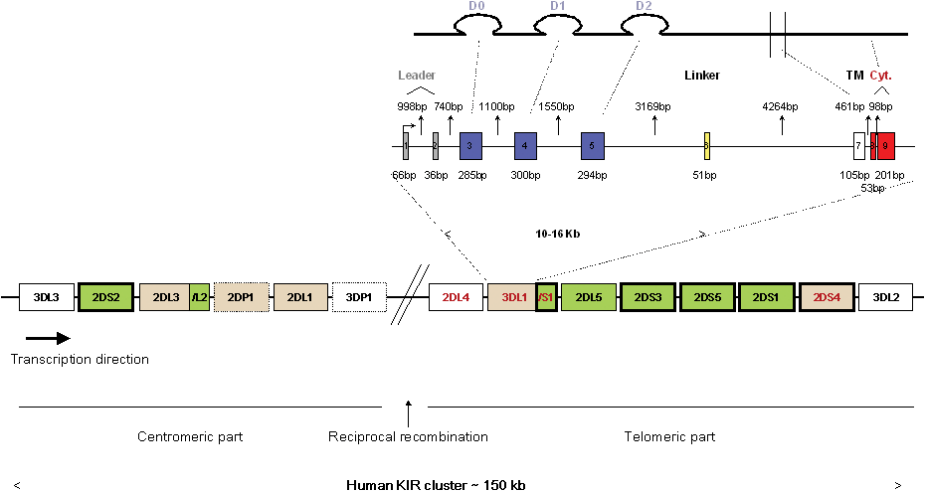
Organisation of the human KIR cluster. The *Killer-cell Immunoglobulin-like receptor (KIR)* genes are organised in a head-to-tail fashion on human chromosome region 19q13.4. Each gene has nine exons (illustrated for *KIR3DL1*) and is roughly 10–16 kb in length, with short and equally homologous intergenic sequences of about 2 kb separating each pair of genes. The organisation of the exon-intron structure of the different *KIR* genes is fairly consistent with the following basic arrangement: the signal sequence is encoded by the first two exons (in grey), each Ig domain (D0, D1, D2) corresponds to a single exon (exons 3–5, respectively, in blue), the linker (in yellow) and transmembrane (in white) regions are each encoded by a single exon (exons 6 and 7, respectively), and the cytoplasmic domain is encoded by two final exons (8 and 9, in red). Two systems have been generated for naming *KIR* genes. The first follows the CD nomenclature system as CD158a, CD158b, etc., based on an approximate centromerictelomeric order of the genes on chromosome 19 [58]. As the CD nomenclature is not used routinely since it does not reflect structure, function, expression or localization, the Human Genome Organization (HUGO) nomenclature will be used throughout this report (www.gene.ucl.ac.uk/nomenclature/genefamily/kir.html). The HUGO nomenclature system, accounts for KIR protein structure and consists of four major subdivisions based on two features: the number of extracellular Ig domains (2D or 3D) and the length of the cytoplasmic tail (L: long or S: short). This latter information determines their functions: inhibitory (L) or activating (S). The boxes in bold indicate the activating *KIR* genes, and the dotted boxes correspond to the pseudogenes. The two boundary genes of the *KIR* cluster, *KIR3DL3* and *KIR3DL2,* and the centrally situated *KIR3DP1* pseudogene and *KIR2DL4* are present in almost all individuals and are therefore considered to be framework (or anchor) genes (shown in white) [59]. *KIR3DL3* and *KIR3DP1* delimit the centromeric part of the *KIR* locus, whereas *KIR2DL4* and *KIR3DL2* delimit the telomeric part. A 14 kb stretch of unique sequence separating *KIR3DP1* from *KIR2DL4* is the preferred site for reciprocal recombination, a mechanism resulting in the formation of new haplotypes by the reassortment of centromeric and telomeric genes. Apart from these framework genes, *KIR* gene content is highly variable in terms of both the number and type of genes present. Although initially considered to be separate genes, *KIR2DL2* and *KIR2DL3* segregate as alleles of the same locus. Similarly, *KIR3DS1* segregates as an allele of the inhibitory *KIR3DL1*. Overall, there are nine variable genes—*KIR2DS2, KIR2DL2/2DL3, KIR2DL1, KIR3DS1/3DL1, KIR2DL5, KIR2DS3, KIR2DS5, KIR2DS1, KIR2DS4*—and one variable pseudogene (*KIR2DP1*). *KIR2DL5* *KIR2DS3* and *KIR2DS5* may be found in both parts of the locus. We decided to represent these genes in the telomeric part of the cluster in this figure. Two major *KIR* haplotype groups, A and B, are classically described. The A haplotype is defined as containing the *KIR3DL3, KIR2DL3, KIR2DL1, KIR2DL4, KIR3DL1, KIR2DS4* and *KIR3DL2* genes. *KIR* gene polymorphism adds further diversity to the *KIR* region, with multiple alleles known for each *KIR* locus. Allele identification was performed for *KIR2DL4, KIR2DS4* and *KIR3DS1/3DL1* (indicated in red). This makes it possible to subdivide haplotype A further, according to the presence of the two common forms of *KIR2DS4*—the wild type *KIR2DS4wt* (haplotype A1) or the deletion variant *KIR2DS4del,* identical to *KIR2DS4wt* except for a 22 base-pair deletion causing a frame shift in translation (haplotype A2). B haplotypes are more variable and are characterised by the presence of more than one activating *KIR* gene. Genes that can be present in both group A and group B *KIR* haplotypes are shown in brown, and genes and/or alleles specific to group B *KIR* haplotypes are shown in green.

The human *Killer cell Immunoglobulin-like receptor* (*KIR*) genes correspond functionally to the murine *Ly49* gene family, providing a potential example of convergent evolution [24]. KIRs are inhibitory or activating transmembrane receptors, present on the surface of NK cells [25]. The length of their intracytoplasmic domains—long (KIR-L), or short (KIR-S)—determines whether they are inhibitory (KIR-L) or activating (KIR-S). Inhibitory KIRs are essential for the regulation of NK cell function via interactions with MHC class I molecules [26]. The ligands of activating KIRs are less well known, although recent results have suggested that activating and inhibitory receptors recognise the same sets of peptide-MHC class I complexes, but differ in binding affinities [27]. The common Human Genome Organization (HUGO) nomenclature for KIRs will be used throughout this report (Figure 1). The HUGO nomenclature accounts for KIR protein structure and consists of four major subdivisions based on two features: the number of extracellular Ig domains (2D or 3D) and the length of the cytoplasmic tail (L or S). The KIRs are encoded by a cluster of genes (Figure 1) varying considerably between individuals, resulting in the expression of between seven and 12 receptors [26]. *KIR* gene polymorphism adds further variability, with multiple alleles identified for each *KIR* locus, but the principal source of diversity in the *KIR* region remains the presence/absence of *KIR* genes. Several case/control studies have reported an association between the presence/absence of specific *KIRs* and disease progression following infection with some oncogenic viruses [25]. For example, the presence of activating *KIR3DS1* in combination with its HLA class I ligand (the *Bw4I80* allele) has been shown to protect against progression to acquired immunodeficiency syndrome in HIV-infected patients [28], and against the development of hepatocellular carcinoma in hepatitis C virus-infected patients [29]. However, the presence of this gene has also been associated with an increase in the risk of human papilloma virus-induced cervical cancer [30]. Consistent with this finding, patients with EBV-associated nasopharyngeal carcinoma (NPC) tend to have more activating *KIRs* than controls [31].

Alltogether these data suggest that KIRs may be involved in the pathogenesis of virus-induced malignancies, although the role of activating and inhibitory receptors remains unclear. In the present work, we investigate the role of KIRs in the occurrence of HL. Our main study was based on a familial design, presenting two major advantages. First, we were able to use full genotypic information for *KIR* loci (number of gene copies), with the corresponding haplotypes, rather than the phenotypic information (presence/absence of the gene) used in previously published case/control designs investigating the association of KIRs with other virus-induced diseases. Second, family-based association studies are not subject to the possible confounding of gene-phenotype associations due to inappropriately chosen controls or population substructures. Our results for this familial sample show that some activating *KIRs* have a protective effect against HL, although this association was not reproduced in a tentative replication case/control study.

## Results

### KIR gene content in HL patients

**Figure 2:**
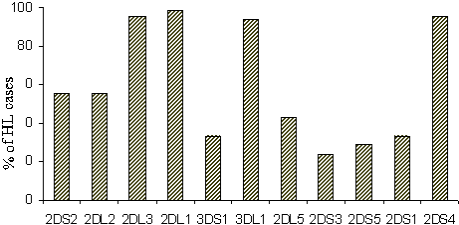
Phenotypic distribution of KIRs in Hodgkin's lymphoma cases. The proportion (percentages) of present *KIR* genes, as determined by *KIR* gene content analysis are presented for the index HL cases (n = 90). Eleven *KIR* genes are presented in the figure. Two pairs of *KIR* genes are allelic: *KIR3DL1/3DS1,* and *KIR2DL2/2DL3*.

Our sample consisted of 90 nuclear families, including 345 subjects (90 index HL cases, 2 affected parents, 143 unaffected parents, 3 affected sibs and 107 unaffected sibs). The mean age at diagnosis of the 90 index cases of HL was 26.2 years (range 16 to 35 years). The index cases comprised 51 men (57%) and 39 women (43%). The main morphological group was nodular sclerosis (87%), followed by mixed cellularity (10%). Figure 2 shows the distribution of the presence of the variable *KIRs*, as determined by *KIR* gene content analysis for the HL index cases (n = 90). The proportion of HL cases with a positive KIR phenotype varied from 23% for *KIR2DS3* to more than 95% for *KIR2DL1*. Standard *KIR* gene content analysis provides only binary phenotypic results for each *KIR* (absent or present), with no information about the number of copies/alleles (1 or 2) when the *KIR* is present. As our analysis focuses on the genotypes (number of copies) of each of the nine variable *KIR* genes shown in figure 1 (*KIR2DS2, KIR2DL2/2DL3, KIR2DL1, KIR3DS1/3DL1, KIR2DL5, KIR2DS3, KIR2DS5, KIR2DS1, KIR2DS4*), we first reconstructed the genotypes from the *KIR* gene content results.

### Genotype reconstruction in families

**Figure 3:**
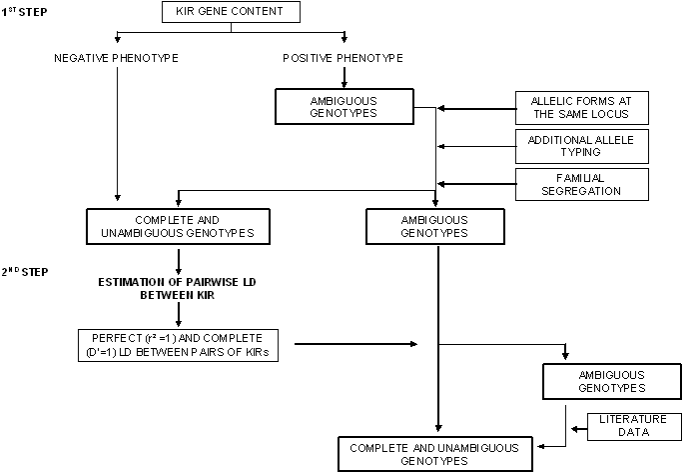
Genotypic reconstruction. *KIR* gene content analysis provides only binary phenotyping results for each KIR (absent or present), rather than complete genotypic information. Thus, for genotype analysis (i.e. the number of copies of each *KIR* gene), genotypic reconstruction in two successive steps was required. The first step combined the results of *KIR* gene content analysis, knowledge of allelic forms of *KIRs*, the additional allele typing carried out for three *KIR* genes, and the analysis of intra-familial segregation. *KIR* gene content made it possible to determine genotype unambiguously only in individuals with a negative phenotypic result. For example, an individual with a negative *KIR2DS2* phenotype has no copy of the gene, and its genotype can be deduced and denoted *KIR2DS2*(−,−). Conversely, an individual with a positive phenotype for *KIR2DS2* may have either one copy of the gene, denoted *KIR2DS2*(+,−), or two copies of the gene, denoted *KIR2DS2*(+,+). Two of the nine variable *KIR* genes have known allelic forms: *KIR2DL2/2DL3* and *KIR3DS1/3DL1*. We defined as (+,+) the *KIR3DS1/3DL1* genotype of subjects homozygous for the presence of *KIR3DS1* (phenotype *KIR3DS1*+, *KIR3DL1*−) and as (−,−) the genotype of subjects homozygous for the absence of *KIR3DS1* (phenotype *KIR3DS1*−, *KIR3DL1+*). An individual with the *KIR3DS1*+, *KIR3DL1*+ phenotype has one copy of each gene and is therefore heterozygous at the locus, with genotype *KIR3DS1/3DL1*(+,−). The genotypes of *KIR2DL2/2DL3* followed the same principle. Additional *KIR* allele typing was especially useful for the analysis of familial gene segregation*.* In particular, for *KIR2DS4,* it made it possible to distinguish between the two common forms, *2DS4wt* and *2DS4del* (Figure 1). The second step of the reconstruction was based on the *KIR* haplotype determination of individuals in each family. We first estimated the pairwise linkage disequilibrium (LD) pattern between *KIR* genes in our families, using the genotypic information obtained from the first step of reconstruction (see statistical analysis). Perfect and complete LD between pairs of *KIRs* made it possible to infer certain genotypes. For the few remaining unsolved *KIR* genotypes, haplotypic reconstruction was based on previously described haplotypes [26], [33].

The procedure used for genotypic reconstruction is described in detail in Figure 3. In the first step, genotypes could be unambiguously determined for all negative KIR phenotypes, and for the two *KIR* genes with known allelic forms, *KIR2DL2/2DL3* and *KIR3DS1/3DL1*. Allele typing for *KIR2DS4* distinguished between the two forms—*KIR2DS4wt* and *KIR2DS4del—*making it possible to reconstruct most of the genotypes for this receptor. Finally, an analysis of familial segregation provided additional genotype determination, resulting in 60% to 100% of the genotypes being known (Table 1), for all *KIRs* except *KIR2DL1*. The very small number of negative subjects (10/345) and the absence of additional information for *KIR2DL1* made it impossible to distinguish between heterozygous *KIR2DL1* (+,−) and homozygous *KIR2DL1* (+,+) in most families. Based on both genotypic reconstitution and additional allele typing of the anchor gene *KIR2DL4*, two families with Mendelian inconsistencies were excluded. Four additional families were also excluded, as individuals did not fit the pattern of familial segregation at two adjacent loci, *KIR2DL4* and *KIR3DS1/3DL1*, due either to Mendelian incompatibilities or to possible deletion of these loci in one haplotype. Finally, the sample subsequently used for linkage disequilibrium (LD) and association studies consisted of 84 families, comprising 322 subjects and including a total of 88 HL patients.

**Table 1:** Summary of the genotypic reconstruction results for the nine variable *KIR* genes in 322 subjects. The procedure of reconstruction is detailed in figure 3.

*KIR*	*2DS2*	*2DL2/2DL3*	*2DL1*	*3DS1/3DL1*	*2DL5*	*2DS3*	*2DS5*	*2DS1*	*2DS4*
Patients with known genotype:									
-after the 1^st^ step N (%)	216 (67)	322 (100)	27 (8)	322 (100)	231 (72)	285 (89)	280 (87)	268 (83)	193 (60)
-after the 2^nd^ step N (%)	322 (100)	322 (100)	27 (8)	322 (100)	320 (99)	321 (100)	322 (100)	322 (100)	322 (100)
Allele frequency^a^	0.33	0.33/0.67	-b	0.25/0.75	0.29	0.15	0.19	0.23	0.77

a estimated from all the reconstructed genotypes after the 2^nd^ step.

b excluded after the second step of reconstruction.

**Figure 4:**
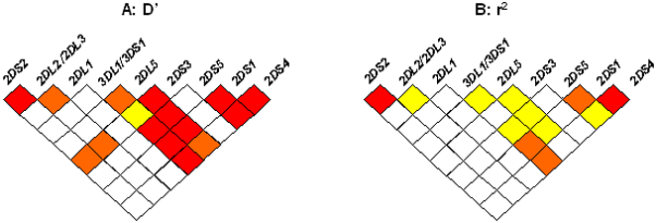
Pairwise linkage disequilibrium between *KIR* genes in the family sample. This analysis was based on the genotypes reconstructed after the first step, without using any information about LD pattern between *KIR* genes. Each square represents the magnitude of pairwise LD between *KIR* genes, as measured by D' (panel A) or r^2^ (panel B). Red squares represent a |D'| or r^2^ value of 1.0, corresponding to complete and perfect LD, respectively. Orange, yellow, and white squares indicate |D'| or R^2^ values of [0.75-1[, [0.5-0.75[, and<0.5, respectively.

We then estimated pairwise LD between *KIRs* based on the genotypes determined in this first reconstruction step (Figure 4). As previously reported, the *KIR* region was in strong LD, and two pairs of *KIRs* were found to be in perfect LD (r^2^ = 1). *KIR2DS1* and *KIR2DS4* were in perfect negative LD—the presence of one gene in a given haplotype excluding the presence of the other, with only two haplotypes, *KIR2DS1+/KIR2DS4*- and *KIR2DS1-/KIR2DS4*+, observed. *KIR2DL2* and *KIR2DS2* were in perfect positive LD—with only the *KIR2DL2+/KIRDS2*+ and *KIR2DL2-/KIRDS2*- haplotypes observed. These findings, which are fully consistent with previously reported haplotypes [26], made possible the complete genotypic determination of these four *KIRs*. Three of the four remaining *KIRs* that were not fully reconstructed, *KIR2DL5*, *KIR2DS3* and *KIR2DS5*, presented complete LD (|D'| = 1) with several other *KIRs* (Figure 4). Based on these observations, genotypes at these three *KIRs* could be determined for most subjects. Reconstruction of the few remaining unknown *KIR2DL5, KIR2DS3* and *KIR2DS5* genotypes was based on previously reported haplotypes [26], and led to the genotypic determination of all but two individuals for *KIR2DL5* and all but one individual for *KIR2DS3*. By contrast, substantial genotypic reconstruction was not possible for *KIR2DL1*, which was not in strong LD with other *KIRs*. As *KIR2DL1* was not expected to be informative for the association study because of its very high phenotypic frequency (>95%), this receptor was excluded from further analysis.

After these two reconstruction steps, study subjects had known genotypes for eight *KIR* gene systems—*KIR2DS2, KIR2DL2/2DL3, KIR3DS1/3DL1, KIR2DL5, KIR2DS3, KIR2DS5, KIR2DS1*, and *KIR2DS4*—which were used for the family-based association study (Table 1). All these *KIR* gene systems were diallelic (+,−), except *KIR2DS4*, which was triallelic (+wt, +del, and−). Allelic frequencies ranged from 0.15 for *KIR2DS3+* to 0.77 for *KIR2DS4*+ (0.58 for *KIR2DS4del* and 0.19 for *KIR2DS4wt*). The eight *KIR* gene systems were in Hardy-Weinberg equilibrium. The frequencies of the three main *KIR* haplotypes, A1, A2 and B, estimated from these family data were 0.46, 0.12, and 0.42, respectively, consistent with the haplotype A and B frequencies reported in other populations of European origin [32], [33].

### Association study of HL with KIR genotypes

The results of the family-based study of association between HL and the eight *KIR* gene systems are presented in Table 2, assuming a dominant mode of inheritance, which gave the best fit in conditional logistic regression analysis. Alleles *KIR3DS1*+, *KIR2DL5*+, *KIR2DS5*+and *KIR2DS1*+were significantly associated (p<0.05) with a protective effect against HL whereas allele *KIR2DS4del* was associated with an increase in the risk of HL. The strongest effects were observed for alleles *KIR3DS1*+(p<0.006), and *KIR2DS1*+ (p<0.01). Under a model of dominant inheritance, the odds ratio (OR) for developing HL in subjects homozygous *KIR3DS1*(+,+) or heterozygous *KIR3DS1*(+,−) with respect to subjects homozygous *KIR3DS1*(−,−) was 0.44 [95% confidence interval: 0.23–0.85]. Similarly, the OR for *KIR2DS1*(+,+) or *KIR2DS1*(+,−) subjects with respect to *KIR2DS1*(−,−) subjects was 0.42 [0.21–0.85].

**Table 2:** Results of the family-based association study between *KIR* genes and Hodgkin's lymphoma using a dominant model

KIR	frequency present allele	N informative families^a^	p-value^b^	Odds Ratio [95% CI]^c^	Effect
*2DS2*	0.33	37	0.89	1.13 [0.55–2.34]	_
*2DL2/2DL3* d	0.33/0.67	37	0.89	1.13 [0.55–2.34]	_
*3DS1/3DL1* d	0.25/0.75	41	0.006	0.44 [0.23–0.85]	*protective*
*2DL5*	0.29	41	0.02	0.56 [0.30–1.04]	*protective*
*2DS3*	0.15	37	0.11	0.65 [0.34–1.25]	_
*2DS5*	0.19	31	0.05	0.49 [0.23–1.04]	*protective*
*2DS1*	0.23	37	0.01	0.42 [0.21–0.85]	*protective*
*2DS4del* e	0.58	30	0.03	2.22 [0.94–5.27]	*at risk*
*2DS4wt* f	0.19	30	0.67	1.02 [0.50–2.1]	_

a informative families are those with at least one heterozygous (+,−) parent for the corresponding KIR gene.

b computed using the FBAT software.

c computed by conditional logistic regression.

d association results for allelic forms of the same KIR locus are provided for the first variant of the pair.

e deleted form

f wild type

As the five *KIR* genes significantly associated with HL were in strong LD (Figure 4), the next step was to carry out both multivariate and haplotype analyses. Multivariate conditional logistic regression demonstrated that the association between HL and the *KIR* region was supported by a single signal. Once one of the five associated *KIR* genes was entered in the regression model, particularly for the most significant system, *KIR3DS1/3DL1,* the other four genes ceased to have a significant effect. Haplotype analysis provided further detail. It showed that, due to LD, the five associated *KIR* genes were organised into only five common haplotypes (frequency>0.02), accounting for 96% of the haplotypes observed in our population (Table 3). The two haplotypes significantly associated with a protective effect against HL (H2 and H5 in Table 3) contained the same alleles for *KIR3DS1 (+), KIR2DL5 (+)*, *KIR2DS1 (+)*, and *KIR2DS4 (−)*, but different alleles for *KIR2DS5* (+ and −, respectively). We also noted that the two protective haplotypes H2 and H5 did not carry *KIR2DS4*, whereas the risk-associated haplotype (H1) carried the deleted form, resulting in the production of a truncated protein predicted to have no functional effect [34], [35]. These observations made *KIR2DS5* and *KIR2DS4* unlikely candidates for the observed effect. Finally, haplotypes H3 and H4 which carried different *KIR2DL5* alleles, seemed to have opposite effects, although this difference was not significant. Thus, these results suggest that the protective effect against HL is mediated by the presence of *KIR3DS1* or *KIR2DS1* or both genes.

**Table 3:** Association study based on haplotypes constructed from five KIR genes

No	*3DS1*	*2DL5*	*2DS5*	*2DS1*	*2DS4*	Estimated frequency	N fam	p-value	Effect
H1	-	-	-	-	del^a^	0.55	31	0.04	*at risk*
H2	+	+	+	+	-	0.15	29	0.03	*protective*
H3	-	-	-	-	wt^b^	0.15	30	0.54	_
H4	-	+	-	-	del^a^	0.06	16	0.56	*_*
H5	+	+	-	+	-	0.05	11	0.03	*protective*

Analysis performed with the FBAT software using a dominant model

a deleted form

b wild type

We then investigated whether the association between *KIR3DS1, KIR2DS1* and HL was influenced by the HLA class I status of the patients, according to their known ligand specificity. For *KIR3DS1,* we considered the HLA–B genotype, as determined by the *Bw4I80* allele and, for *KIR2DS1*, we considered the HLA–C genotype, as determined by the *C2* allele. No significant heterogeneity (p>0.79) was found in the association between *KIR3DS1* and HL, according to the presence or absence of the *Bw4I80* allele (Table 4). For *KIR2DS1*, the protective effect seemed to increase with the number of *HLAC2* alleles, although the heterogeneity test was not significant (p = 0.35) (Table 4). The OR decreased from 0.52 [0.17–1.65] for *C1/C1* homozygotes to 0.37 [0.15–0.90] for subjects with at least one *C2* allele. Interestingly, when we considered the presence of both ligands (i.e. patients with at least one *Bw4I80* allele and one *C2* allele), the protective effect of *KIR2DS1* and *KIR3DS1* tends to be stronger with an OR of 0.21 [0.04–1.07] for both *KIR* alleles. However, the number of informative families in this latter analysis was small (seven families), precluding to draw definitive conclusion from this result. Finally, we investigated the role of some of the characteristics of EBV-infected patients, focusing on the EBV status of the tumour (positive/negative) or EBV load in peripheral blood mononuclear cells (detectable/undetectable). No significant heterogeneity in the association between HL and *KIR3DS1* or *KIR2DS1* was found for any of these characteristics (Table 4). However, the protective effect seemed to be stronger in patients with EBV-positive tumours or detectable viral load. This effect was clearest for *KIR3DS1*, with an OR of 0.27 [0.08–0.87] for patients with detectable viral load (40% of patients) versus 0.61 [0.27–1.37] for those with no detectable viral load (60%).

**Table 4:** Effect of *KIR3DS1* and *KIR2DS1* stratified on HL patients characteristics

	*KIR3DS1*			*KIR2DS1*		
HL cases	N informative families	Odds-Ratio [95% CI]^a^	p^b^	N informative families	Odds-Ratio [95% CI]^a^	p^b^
All	41	0.44 [0.23–0.85]	0.006	37	0.42 [0.21–0.85]	0.01
*HLA-B*						
No *Bw4I80* allele	30	0.46 [0.21–1.01]	0.01	-	-	-
At least one *Bw4I80* c	11	0.38 [0.11–1.31]	0.24	-	-	-
*HLA-C*						
* C1/C1*	-	-	-	13	0.52 [0.17–1.65]	0.23
* *At least one *C2*	-	-	-	24	0.37 [0.15–0.90]	0.02
*HLA-B* and *-C* combined						
* C1/C1* or no *Bw4I80*	34	0.51 [0.25–1.06]	0.04	30	0.52 [0.23–1.10]	0.10
* *At least one *Bw4I80* and one *C2*	7	0.21 [0.04–1.07]	0.03	7	0.21 [0.04–1.07]	0.03
EBV *in situ* d						
EBV+	8	0.24 [0.05–1.24]	0.09	7	0.28 [0.05–1.51]	0.14
EBV−	22	0.37 [0.14–0.97]	0.02	19	0.37 [0.13–1.04]	0.04
EBV load in PBMCse, f						
detectable	16	0.27 [0.08–0.87]	0.02	15	0.28 [0.09–0.92]	0.03
undetectable	24	0.61 [0.27–1.37]	0.15	22	0.54 [0.22–1.23]	0.17

a computed by conditional logistic regression

b computed by the FBAT software under a dominant model

c at least one present allele. Only two HL patients are *Bw4I80* homozygotes.

d missing in 11 cases

e missing in 1 case

f PBMCs corresponds to peripheral blood mononuclear cells

We tried to replicate these results by comparing KIR phenotype frequencies in the independent sample from the ENGELA study consisting of 68 HL patients and 60 controls. In this sample, no significant association was observed between any KIR phenotype and HL. Further analyses stratified for age (e.g. restricted to the 15–35 age class), HLA class I genotype, and the EBV status of tumour tissues identified no HL subgroup associated with *KIR3DS1* or *KIR2DS1*. In this sample, the frequencies of positive phenotypes for KIR3DS1 and KIR2DS1 among HL cases were 43% (29/68) and 41% (28/68), respectively. These frequencies were higher than for the 84 index HL cases of the familial study (33% for both KIR phenotypes), although these differences were not significant (p = 0.27 and 0.24 respectively).

## Discussion

We report here the first family-based association study to show an association between a disease and genes of the *KIR* cluster. Familial studies have been conducted to determine the structure of the *KIR* region [26], [36], but the previously reported associations between the presence/absence of *KIRs* and various disease phenotypes were identified in case/control studies [25]. Family-based association studies have important advantages over case/control studies in that they can make use of full genotypic information at *KIR* loci (number of gene copies) and make it possible to avoid the possible confounding of gene-phenotype associations due to inappropriately chosen controls or population admixture. A first phase of genotypic reconstruction is required, based principally on a combination of information from the familial data. Published data were required for the full determination of only a minority of genotypes (for *KIR2DL5, KIR2DS3* and *KIR2DS5*), and *KIR2DL1* was the only locus that could not be reconstructed, due to its high frequency. We propose a method of *KIR* genotypic reconstruction in familial samples that could be applied to other family-based studies, as already demonstrated for a sample of African origin (S.R. *et al*., unpublished data).

Host genetic factors are known to be important in HL pathogenesis [4]–[7], [11], and many association studies have suggested a possible association between HL and HLA genes [8]–[10]. Given the strong link between EBV and HL and the role of HLA in the presentation of viral antigens, EBV specificities in the role of HLA genes in HL have recently been investigated. An association was found between EBV-positive HL and markers located in the HLA class I region, whereas EBV-negative HL was found to be associated with markers of the HLA class III region [10]. However, no consensus has yet emerged on the role of any specific HLA molecule [8]–[10]. KIRs were considered as candidate genes for HL predisposition in this study since they are alternative receptors for HLA class I molecules.

Our family-based analysis provides the first evidence for an association between HL and certain *KIR* alleles in strong linkage disequilibrium. Refined haplotype analysis results were consistent with a dominant protective effect of *KIR3DS1* and/or *KIR2DS1* against HL. The validity of our familial analysis is also strengthened by the observation of random transmission of the *KIR* alleles considered in the 100 unaffected children of our sample (i.e. the healthy siblings of HL patients). We tried to replicate this association, using a case/control sample collected in the context of a French survey on adult lymphoma and including 68 HL patients. No significant association between HL and the presence/absence of any *KIRs* was found. The HL patients did not differ between the two samples in terms of ethnic origin and clinical features. EBV viral load in peripheral blood mononuclear cells was not determined in the case/control study, whereas we found that the protective effect of *KIR3DS1* and/or *KIR2DS1* in familial sample tended to be stronger in patients with detectable viral load. We also restricted the analysis to the 34 patients belonging to the same age class (15–35 years) as our familial patients, corresponding to the first peak of HL incidence in developed countries, but no significant association was observed in this small sample. In any case, a larger sample is certainly required for a more powerful replication study.

Due to strong linkage disequilibrium, it was almost impossible to distinguish between the effects of *KIR2DS1* and *KIR3DS1*. The observed trend for the protective effect of *KIR2DS1* to increase in the presence of its putative ligand, HLA-C2, may be more consistent with a role for this receptor although the effectives of each strata are small. We cannot exclude the possibility that both these KIRs are responsible for the observed protective effect, as different cognate KIR-HLA class I pairs may have the same biological effects, since, for example, all activating KIR-S share the same KARAP/DAP12-dependent signalling pathways [37]. This hypothesis is supported by the trend toward a stronger protective effect in presence of at least one allele of each HLA class I ligands of *KIR3DS1* and *KIR2DS1*, and the even greater trend in presence of both ligands. Our results are consistent with those of a study showing the protective effect of *KIR3DS1* against HCV-related hepatocarcinoma [29], and with the general idea that activating KIRs are beneficial for the response to infectious diseases and tumours. However, immune activation may not be beneficial at all stages of these disorders and could, for example, increase the risk of other tumours associated with local inflammation [25]. This may account for two other reports of activating KIRs being associated with an increase in the risk of virus-related localised cancers, such as NPC [31] and cervical cancer [30].

We observed an interesting trend towards a stronger protective effect of *KIR2DS1* and/or *KIR3DS1* in the presence of EBV, detected in either HL tumour cells or in peripheral mononuclear blood cells. This suggests that the protective role of activating KIRs may be more pronounced in HL cases associated with EBV. NK cells were recently shown to limit lytic EBV infection through the down-regulation of MHC class I molecules that bind to inhibitory KIRs, and up-regulation of activating ligands such as ULBP-1 and CD112 [38]. An indirect control of virus replication by cytokines produced by NK cells may also be involved in this protective effect, as shown for mouse cytomegalovirus [39]. We favor another model for the potential role of NK cells in the control of HL via activating KIRs. The malignant Hodgkin/Reed-Sternberg (HRS) cells of EBV-associated HL display high-level expression of MHC class I molecules [12], [13], [41]. The up-regulation of HLA class I-peptide complexes in HRS cells would then trigger NK cell effector functions via activating KIRs, leading to the participation of NK cells in the control of HL. This hypothesis is consistent with previous biological findings as *KIR2DS1* has been shown to recognize peptide-HLA class I complexes that are up-regulated on EBV human infected B cells [27]. Similarly, the control of mouse cytomegalovirus infection in certain strains of mice involves the recognition of peptide-MHC class I complexes through the activation of Ly49 receptors [40]. To further confirm our hypothesis, it will be of particular interest to follow the activation of NK and T cell subsets according to the presence of *KIR2DS1* and/or *KIR3DS1* when the detection of these receptors will be possible. In any case, the recognition of HL cells by KIRs warrants further investigation, and we are currently carrying out an immunohistological study, comparing the presence of NK cells in EBV-positive and EBV-negative HL specimens.

## Methods

### Study population

HL index cases were recruited from three haematology units in the Paris area. Patients were included at diagnosis (n = 21) or whilst in complete remission, at least one year after diagnosis (n = 69). Inclusion criteria were age between 15 and 35 years and negative serological tests for HIV at diagnosis. Tumour biopsies were reviewed by expert haematological pathologists (JB, DC, MR). Intratumoral EBV expression was assessed by mRNA *in situ* hybridisation for Epstein-Barr virus-encoded small non polyadenylated RNA (EBER1) sequences or by indirect immunofluorescence staining for latent membrane protein 1 (LMP1). First-degree relatives (parents and siblings) of the patients were asked to participate in the study. All participating subjects were interviewed to determine their medical history, and all gave a blood sample for DNA extraction and EBV measurements. EBV viral load was measured in peripheral blood mononuclear cells, as previously described [42]. All recruited families (n = 90) included the index HL case and at least two other individuals (either both parents or one parent and one unaffected sibling). Five of the 90 families were multiplex (three families with two affected siblings, and two families with an affected parent and an affected child).

In addition to this family sample, we also studied a case-control sample recruited in the framework of the ENGELA (*Environnement et Génétique des Lymphomes de l'Adulte*) study on adult lymphoma. This study was conducted in six French cities (Brest, Bordeaux, Caen, Lille, Nantes and Toulouse), and included newly diagnosed HIV-negative HL cases confirmed by histology. Controls and cases were matched for centre, sex, age (±3 years) and ethnic origin, with the controls hospitalised or consulting at the same hospitals as the cases, but for reasons other than cancer, occupational diseases or injuries, and tobacco-or alcohol-related diseases. DNA samples were available for 68 Caucasian cases (52 with nodular sclerosis, 11 with mixed cellularity, 5 with HL of another or an unspecified type), including 40 men and 28 women, and 60 Caucasian controls (36 men and 24 women), mostly from orthopaedic departments. The subjects included were aged 18 to 71 years, and 32 cases and 23 controls were less than 35 years old. The EBV status of tumour tissue was available for 46 cases, 13 (28%) of which were positive.

Both studies were approved by the French Consultative Committee for Protecting Persons in Biomedical Research (CCPPRB) of Paris Necker and Kremlin-Bicêtre, respectively. Human experimentation guidelines were followed in the conduct of the research. Informed consent was obtained from adults or from parents of minors.

### Molecular methods

#### KIR gene content

*KIR* gene content was determined with an updated version of the previously reported PCR-SSOP technique [43], which consists in oligonucleotide primers and probes selected from the first *KIR* gene typing system, as described in [44]. Briefly, we used two separate locus-specific PCR to amplify the D1, D2 and transmembrane/cytoplasmic tail regions. The D1 and D2 regions were amplified together, with primers flanking exons 4 and 5; 5′AGAGAXXGTCATCCTGCA(A/G)TGTTGGTC 3′ (exon 4, nucleotides 56-82) and 5′CTCACCTXTGAC(G/A)GAAACAAGCAGTGG 3′ (intron 5-273 exon 5). The transmembrane/cytoplasmic tail region was amplified as previously described. PCR products were blotted onto nylon membranes and hybridised with 19 digoxigenin-labelled sequence-specific oligonucleotide probes, using hybridisation and chemiluminescence procedures, as previously described. Internal control samples, identified with a unique probe pattern, were also included in each experiment.

#### Additional KIR allele typing

*KIR2DL4, KIR2DS4* and *KIR3DS1/3DL1* alleles were identified using previously described SSOP techniques, based on a gene-specific PCR product [36], [45], [46]. *KIR2DL4* allele typing consists involves the PCR amplification of a 1440 bp region of *KIR2DL4* exons 3/5 (D0/D2 domains), followed by hybridisation, using 16 digoxigenin-labelled probes for allele discrimination. The *KIR2DS4* allele typing system is based on the specific amplification of the 199/221bp region of *KIR2DS4* exon 5 (D2 domain) and 11 probes were used for allele identification. Finally, the *KIR3DS1/3DL1* allele typing strategy uses three separate specific amplifications of the extracellular D0 (exons2/3), D1 and D2 domains (exons 4/5) and a section of the cytoplasmic tail (exons 8/9), respectively. The third amplification concerned a single region of *KIR3DS1* only, and was required for discrimination between certain *KIR3DS1* alleles. The amplified DNA was subjected to SSOP typing, using a total of 28 oligonucleotide probes, as previously reported. In each system, a set of nine internal control samples with a unique pattern of probe reactivity was included among the samples investigated. Computational analysis was carried out on the data obtained with the various probes, to identify the alleles of each gene present.

#### HLA class I KIR ligand typing

The study was focused on the identification of HLA-Bw4 and HLA-C1/C2 KIR ligands, that were typed using updated methods, as described in [47]. Briefly, we used four probes (BL20, BL21, BL22, BL23) to define HLA-Bw4, with the same PCR conditions as the previously report typing system for HLA–B [48]. These probes are able to distinguish threonine or isoleucine present at position 80 of the HLA-Bw4 molecules. Similarly, HLA-C1 and C2 groups were defined using only two probes from the full HLA–C allele typing system [49], which differentiated nucleotides at positions 77 and 80. These probes were C293 for C1 group and C291 for C2 group.

### Statistical analysis

Hardy-Weinberg equilibrium was tested after genotypic reconstruction for each *KIR* gene, using all the parents of the families. Measures of pairwise linkage disequilibrium (LD) between *KIR* genes, such as D' and r^2^ [50], were estimated using Haploview (http://www.broad.mit.edu/personl/jcbarret/haplo/). |D'| and r^2^ vary between 0 and 1. Complete LD in a pair, defined as |D'| = 1, is obtained when one of the four possible haplotypes is not observed. Perfect LD between two KIR genes, defined as r^2^ = 1, is reached when two of the four possible haplotypes are not observed, i.e |D'| = 1 and the frequencies of the (+,−) alleles of the two *KIR* genes are equal.

The main association study was conducted on the family sample, using specific methods developed in this context. Family-based association studies avoid the possible confounding of gene-phenotype associations due to inappropriately chosen controls that can occur in case/control studies. The general principle of these studies is to search for a distortion of the transmission of alleles from parents to affected offspring, by means of the “transmission disequilibrium test” (TDT) [51]. Families with one missing parent (30/84 in our sample) can be analysed either by reconstructing parental genotypes from children (RC-TDT) [52], or by using unaffected siblings as controls (Sib-TDT) [53]. The family-based association study was carried out mainly according to the method implemented in the FBAT program [54], which combines these three different methods (TDT, RC-TDT, and Sib-TDT). This method also allows the use of an empirical variance-covariance estimator for the statistic, which is consistent when sibling marker genotypes are correlated (e.g. when the analysis includes multiple affected sibs) [55]. Finally, alleles for which evidence of association was obtained were also analysed by conditional logistic regression, as previously described [56]. This analysis generated odds-ratio (OR) estimates and made it possible to compare different models of dominance (recessive, dominant or additive). It also made it possible to test for heterogeneity of the sample according to a qualitative criterion (e.g. HLA class I genotypes) by performing the analysis on the whole sample, and separately on subsamples, as described elsewhere [57]. For the association study conducted on the case/control sample, KIR phenotype distributions were compared between HL patients and controls, using classical chi-squared tests. Conditional logistic regression analyses (proc PHREG) and chi-squared tests (proc FREQ) were carried out with SAS software (version 8.2, SAS Institute, Cary, NC).
